# Factors related to presenteeism: a focus group interview study with Portuguese and Swiss nurses

**DOI:** 10.1192/j.eurpsy.2023.687

**Published:** 2023-07-19

**Authors:** C. Laranjeira, F. Pereira, H. Verloo, M. Bieri, A. Querido

**Affiliations:** ^1^ School of Health Sciences; ^2^ciTechCare, Polytechnic of Leiria, Leiria, Portugal; ^3^School of Health Sciences, HES-SO Valais/Wallis, Sion, Switzerland

## Abstract

**Introduction:**

Nurse presenteeism has long been of global concern, with impacts on nurse staffing levels, patient care, and hospital costs.

**Objectives:**

This international study aimed to explore the factors associated with presenteeism among frontline nurses and nurse managers in acute, primary, and long-term healthcare settings in Portugal and Switzerland.

**Methods:**

A qualitative descriptive study involving online Focus Groups (FGs). The FGs included 55 participants and lasted 5 months (from March 2021 to July 2021). A purposive sampling strategy was used to select nurses. The inclusion criteria were as follows: (a) working in a public or private healthcare setting with at least one month of experience in their current workplace (which is officially considered the time required for integration); (b) working at least 20% of a full-time equivalent position; and (c) having a bachelor’s, master’s, or PhD degree. This study followed the COREQ checklist.

**Results:**

Participants included 55 nurses: 49 females and 6 males. Three main reasons for presenteeism were identified: unfamiliar terminology; the paradoxical effect of `being present’ but absent; and presenteeism as a survival strategy. Six contributing factors were also recognized: (a) institutional disinterest toward employees; (b) paradigm shift: the tension between person-centered and task-centered care; (c) sudden changes in care practices due to the COVID-19 pandemic; (d) a lack of shared work perspectives with hierarchical superiors; (e) the financial burden of being absent from work; and (f ) misfit of human responses (Laranjeira et al., 2022).

**Conclusions:**

This study has generated in-depth knowledge about concepts and causes of presenteeism and has instructive for a broad audience of nurse managers and leaders. Our thematic analysis shows that presenteeism can be explained by factors related to the pressure to attend work, by individuals’ constraints and commitment and by the organizational environment.

**References:**

Laranjeira, C., Pereira, F., Querido, A., Bieri, M., & Verloo, H. (2022). Contributing Factors of Presenteeism among Portuguese and Swiss Nurses: A Qualitative Study Using Focus Groups. *International journal of environmental research and public health*, *19*(14), 8844.

**Disclosure of Interest:**

None Declared

